# The importance of being integrative: a remarkable case of synonymy in the genus *Viridiscus* (Heterotardigrada: Echiniscidae)

**DOI:** 10.1186/s40851-021-00181-z

**Published:** 2021-11-20

**Authors:** Piotr Gąsiorek, Katarzyna Vončina, Diane R. Nelson, Łukasz Michalczyk

**Affiliations:** 1 Department of Invertebrate Evolution, Institute of Zoology and Biomedical Research, Faculty of Biology, Jagiellonian University, Gronostajowa 9, 30-387 Kraków, Poland; 2 Department of Biological Sciences, East Tennessee State University, Johnson City, TN 37614 USA

**Keywords:** DNA barcoding, Dorsal sculpturing, Integrative taxonomy, Junior synonym, Nearctic

## Abstract

There are two predominant sources of taxonomically useful morphological variability in the diverse tardigrade family Echiniscidae: the internal structure and surface sculpture of the cuticular plates covering the dorsum (sculpturing) and the arrangement and morphology of the trunk appendages (chaetotaxy). However, since the appendages often exhibit intraspecific variation (they can be reduced or can develop asymmetrically), sculpturing has been considered more stable at the species level and descriptions of new echiniscid species based solely on morphology are still being published. Here, we present a case study in which a detailed analysis of the morphology and multiple genetic markers of several species of the genus *Viridiscus* shows that cuticular sculpture may also exhibit considerable intraspecific variation and lead to false taxonomic conclusions. In a population collected from the eastern Nearctic, in the type locality of the recently described species *V. miraviridis*, individuals with transitional morphotypes between those reported for *V. viridissimus* and *V. miraviridis* were found. Importantly, all morphotypes within the *viridissimus*–*miraviridis* spectrum were grouped in a single monospecific clade according to rapidly evolving markers (ITS-1, ITS-2 and COI). Given the morphological and genetic evidence, we establish *V. miraviridis* as a junior synonym of *V. viridissimus*. This study explicitly demonstrates that a lack of DNA data associated with morphological descriptions of new taxa jeopardizes the efforts to unclutter tardigrade systematics. Additionally, *V. perviridis* and *V. viridissimus* are reported from Lâm Đồng Province in southern Vietnam, which considerably broadens their known geographic ranges.

**Supplementary Information:**

The online version contains supplementary material available at 10.1186/s40851-021-00181-z.

## Introduction

In contrast to anatomically simplified eutardigrades, heterotardigrades usually exhibit complex morphology that is presumably closer to the ancestral tardigrade morphotype (e.g., [1–3]). Both marine and limnoterrestrial heterotardigrades are characterized by the presence of cephalic and trunk appendages [4, 5]. Their cuticle is often highly sculptured, comprising endocuticular pillars [6], variously shaped protrusions [7–9] and/or pores in the epicuticular layer (e.g., [10, 11]). Historically, tardigradologists had focused on differences in chaetotaxy when establishing new taxa, leading to the formation of problematic species complexes, such as the *Echiniscus blumi–canadensis* group [12]. More recently, improved microscopy tools have allowed the detailed interpretation of the sculpturing of the dorsal plates (e.g., [13]), which has emerged as an equally important factor in taxonomic, systematic and phylogenetic reasoning [14, 15].

*Viridiscus* is an unappendaged (*sensu* [15]) genus of echiniscids that displays green to almost black body coloration and composite sculpturing, comprising a dense endocuticular sponge layer and flat epicuticular granules [15–18]. In the redescription of the type species *Viridiscus viridis* [19], Pilato et al. [18] highlighted morphological differences in dorsal sculpturing between the known representatives of *Viridiscus*. Last year, a new Nearctic species, *Viridiscus miraviridis,* was described based on its extraordinarily developed epicuticular layer forming sclerotized ridges, a character previously unknown in the genus [20]. However, attempts to obtain DNA barcode data were unsuccessful, so the original description was based solely on morphological characters. Thus, to amend the description and pinpoint the phylogenetic position of *V. miraviridis*, we sequenced five genetic markers, including four nuclear (18S rRNA, 28S rRNA, ITS–1 and ITS–2) and one mitochondrial (COI) marker, of topotypic specimens of the species and of *Viridiscus viridissimus* [21] and *Viridiscus* aff. *viridianus* [17], which we also found in a moss sample from Tennessee. For comparative purposes, we sequenced additional *Viridiscus* specimens from Madeira and Vietnam.

Unexpectedly, we found that despite clear morphological differences between *V. viridissimus* and *V. miraviridis*, individuals representing both morphotypes shared the same DNA barcodes, and their conspecificity was confirmed by three species delineation methods (ABGD, [22]; bPTP, [23]; and ASAP, [24]). This discovery gave us an opportunity to discuss the currently used taxonomic criteria and note potential problems induced by describing limnoterrestrial tardigrade species without associated genetic barcodes.

## Materials and methods

### Specimens and morphology

Populations of *Viridiscus* were obtained from a total of seven moss samples collected in three locales:

- 32°49′22″N, 16°59′06″W, 321 m asl: **Portugal**, Madeira, Ponta Delgada; moss on asphalt road; coll. Łukasz Michalczyk on 21.02.2018: sample **PT.042**.
- 36°18′N, 82°22′W, 517 m asl: **USA**, Tennessee, Washington County, Johnson City; moss on concrete caps of brick fence posts; coll. Diane Nelson on 07.12.2020: samples **US.077**, **US.078**, **US.080**, and **US.081**.
- 11°56′59″N, 108°25′59″E, 1481 m asl: **Vietnam**, Đà Lạt, Biệt Thự Bạch Dương; moss on stone wall; coll. Daniel Stec in 08.2018: samples **VN.027** and **VN.028**.

All specimens extracted from the samples (using standard methods described in [25]) were subsequently divided into groups used for light microscopy analyses and Sanger sequencing (Table 1). Some specimens were mounted in a small drop of Hoyer’s medium on permanent slides and examined by phase-contrast microscopy (PCM) under an Olympus BX53 light microscope with an Olympus DP74 digital camera at Jagiellonian University. Syntypes of *Viridiscus perviridis* [16], paratypes of *Viridiscus viridianus* [17], and American specimens (slides C.T.7836–63, C.T.7730–43) representing *V. viridissimus* from the Ramazzotti & Maucci collections were examined under a Leica DM RB microscope equipped with a Nikon DS-Fi 1 digital camera in the Department of Life Sciences of the University of Modena and Reggio Emilia (Italy). All figures were assembled in Corel Photo-Paint X8. To obtain clear micrographs of dorsal sculpturing, a series of images were recorded at ca. 0.1 μm intervals of vertical focus and then manually assembled into a single deep-focus image in Corel Photo-Paint 2018.

**Table 1:** List of species and populations used in analyses. Types of analyses: PCM – imaging and morphometry in PCM; DNA – DNA sequencing. The number in each analysis indicates how many specimens were utilized for a given method: ♀ – sexually mature females, ♂ – sexually mature males, j – juveniles, l – larvae, v – vouchers (please note that in some cases, the same specimens were used for both DNA and LCM analyses)

Species	Sample code	Analyses and specimens	
		PCM	DNA
*V. perviridis*	PT.042^a^	5 (1♀ + 4♀v)	4♀
	VN.027^b^	2 (2♀)	0
	VN.028^b^	45 (35♀ + 9j + 1 l)	4♀
*V.* aff. *viridianus*	US.077	1 (1j)	0
	US.078	6 (1♀ + 5♀v)	5♀
	US.081	14 (5♀ + 5j + 4♀v)	4♀
*V. miraviridis*	US.080	2 (1♀ + 1j)	0
	US.078	15 (12♀ + 1♂ + 2♀v)	2♀
	US.081	5 (1♀ + 1♂ + 3j)	0
*V. viridissimus*	US.078	21 (6♀ + 4♂ + 1j + 10♀v)	10♀
	US.080	12 (8♀ + 2♂ + 1j + 1 l)	0
	US.081	17 (7♀ + 3j + 2 l + 5♀v)	20♀
	VN.027^b^	1 (1♀)	0
	VN.028^b^	43 (27♀ + 1♂ + 15j)	4♀

^a^Population also utilized in [15]

^b^The first records of both species from Southeast Asia and the Indomalayan realm

### Genotyping

DNA was extracted from individual animals following a Chelex® 100 resin (Bio–Rad) extraction method [26], with modifications according to [27]. Vouchers (specifically hologenophores) [28] were obtained when possible. Five DNA fragments were sequenced: the 18S rRNA small ribosomal subunit, the 28S rRNA large ribosomal subunit, the internal transcribed spacers ITS-1 and ITS-2, and the cytochrome oxidase subunit I (COI). All fragments were amplified and sequenced according to the protocols described in [27]; the primers and original references for the specific PCR programs are listed in Supplementary Material 1. GenBank accession numbers for all specimens are provided in Table 2. We were not able to obtain COI barcodes for *V. perviridis* (see SM1). All ITS and COI sequences were aligned with sequences of *Echiniscus succineus* as an outgroup using the ClustalW Multiple Alignment tool [29] implemented in BioEdit [30]. The aligned fragments were edited in BioEdit, with gaps left intact in the case of ITS sequences. The alignments are provided as Supplementary Materials 2, 3 and 4. The 18S rRNA and 28S rRNA sequences were not used in developing primary species hypotheses, as they are too conservative [31] and thus not suitable for molecular species discrimination. Nevertheless, since these markers can be used in phylogenetic studies, they are also provided here.

**Table 2:** GenBank identifiers for sequenced *Viridiscus* specimens analyzed in the present study (new sequences are indicated in bold)

Specimen ID	Hologenophore preserved	18S rRNA	28S rRNA	ITS-1	ITS-2	COI
PT.042.03	✓	MK529696	MK529726	**OK094219**	**OK094181**	–
PT.042.04	✓	MK529696	MK529727	**OK094220**	**OK094182**	–
US.078.01	✓	**MZ868191**	**OK094224**	**OK094186**	**OK094148**	**MZ852046**
US.078.02	✓	–	–	**OK094187**	**OK094149**	**MZ852047**
US.078.05	✗	–	–	**OK094188**	**OK094150**	**MZ852062**
US.078.06	✓	**MZ868192**	**OK094226**	**OK094207**	**OK094169**	**MZ852063**
US.078.12	✓	**MZ868197**	**OK094230**	**OK094211**	**OK094173**	**MZ852064**
US.078.13	✓	–	–	**OK094189**	**OK094151**	**MZ852048**
US.078.20	✗	–	–	**OK094190**	**OK094152**	**MZ852049**
US.078.24	✓	**MZ868193**	**OK094227**	**OK094208**	**OK094170**	–
US.078.30	✓	–	–	**OK094212**	**OK094174**	–
US.078.33	✓	–	–	**OK094213**	**OK094175**	**MZ852065**
US.078.37	✓	–	–	**OK094214**	**OK094176**	**MZ852066**
US.081.01	✓	–	–	**OK094191**	**OK094153**	–
US.081.02	✗	–	–	**OK094192**	**OK094154**	–
US.081.04	✓	–	–	**OK094193**	**OK094155**	–
US.081.05	✗	–	–	**OK094194**	**OK094156**	**MZ852050**
US.081.07	✓	**MZ868194**	**OK094225**	**OK094195**	**OK094157**	**MZ852051**
US.081.08	✗	–	–	**OK094196**	**OK094158**	**MZ852052**
US.081.09	✗	–	–	**OK094197**	**OK094159**	**MZ852053**
US.081.10	✗	–	–	**OK094198**	**OK094160**	**MZ852054**
US.081.11	✗	–	–	**OK094215**	**OK094177**	–
US.081.12	✗	–	–	**OK094199**	**OK094161**	**MZ852055**
US.081.13	✗	–	–	**OK094200**	**OK094162**	**MZ852056**
US.081.14	✗	–	–	**OK094201**	**OK094163**	**MZ852057**
US.081.15	✗	–	–	**OK094202**	**OK094164**	**MZ852058**
US.081.16	✓	**MZ868198**	**OK094231**	**OK094216**	**OK094178**	**MZ852067**
US.081.17	✓	–	–	**OK094217**	**OK094179**	**MZ852068**
US.081.18	✓	–	–	**OK094203**	**OK094165**	–
US.081.19	✗	–	–	**OK094218**	**OK094180**	**MZ852067**
US.081.21	✗	–	–	**OK094204**	**OK094166**	**MZ852059**
US.081.22	✗	–	–	**OK094205**	**OK094167**	**MZ852060**
US.081.26	✓	–	–	**OK094206**	**OK094168**	**MZ852061**
VN.028.01	✓	**MZ868195**	**OK094228**	**OK094209**	**OK094171**	–
VN.028.02	✓	**MZ868196**	**OK094229**	**OK094210**	**OK094172**	–
VN.028.03	✓	–	–	**OK094221**	**OK094183**	–
VN.028.04	✓	**MZ868199**	**OK094232**	**OK094222**	**OK094184**	–
VN.028.05	✓	–	–	**OK094223**	**OK094185**	–

### Phylogeny

The sequences of the ITS fragments were concatenated to generate a matrix of 1064 bp in SequenceMatrix [32]. Using PartitionFinder version 2.1.1 [33] with the application of the Bayesian information criterion (BIC) and a greedy algorithm [34], the best substitution model and partitioning scheme were chosen for posterior phylogenetic analysis. As the best-fit partitioning scheme, PartitionFinder suggested the retention of two partitions (I: ITS-1, II: ITS-2), and the best fit model was TVM + G for both partitions; in the case of the COI matrix (611 bp), the best model was TIM + G. Bayesian inference (BI) marginal posterior probabilities were calculated using MrBayes v.3.2 [35]. Random starting trees were used, and the analysis was run for ten million generations, sampling the Markov chain every 1000 generations. An average standard deviation of split frequencies of < 0.01 was used as a guide to ensure that the two independent analyses had converged. Tracer v1.6 [36] was then used to ensure that Markov chains had reached stationarity and to determine the correct burn-in for the analysis (i.e., the first 10% of generations). The effective sample size values were greater than 200, and the consensus tree was obtained after summarizing the resulting topologies and discarding the burn-in. All final consensus trees were viewed and visualized by using FigTree v.1.4.3 available from https://tree.bio.ed.ac.uk/software/figtree.

### Genetic species delineation

MEGA7 version 7.0 [37] was used to calculate uncorrected pairwise distances. Both ITS and COI alignments were uploaded separately to the Assemble Species by Automatic Partitioning (ASAP) web [24] to obtain three independent marker-based primary species hypotheses using uncorrected pairwise distances. The partitions with the lowest ASAP scores and *p* values < 0.05 were chosen as the best-fit hypotheses. In tandem, we applied another phenetic method of species delineation based on genetic distances (automatic barcode gap discovery (ABGD, [22]), with the default options) to the three alignments. Finally, Bayesian Poisson tree processes (bPTP, [23]) were applied to the Bayesian phylogenetic trees of the three markers. In all cases, we discarded the outgroup to protect against eventual biases caused by the distant relationship between the outgroup and ingroup taxa. The calculations were conducted with 100,000 MCMC generations, thinning the set to 100, with 10% burn-in, and with searches for maximum likelihood and Bayesian solutions.

## Results

### Morphology (Figs. 1–2)

Except for samples PT.042 and US.077, all of the other samples analyzed in the present study contained mixed *Viridiscus* morphotypes (Table 1).

**Fig. 1:**
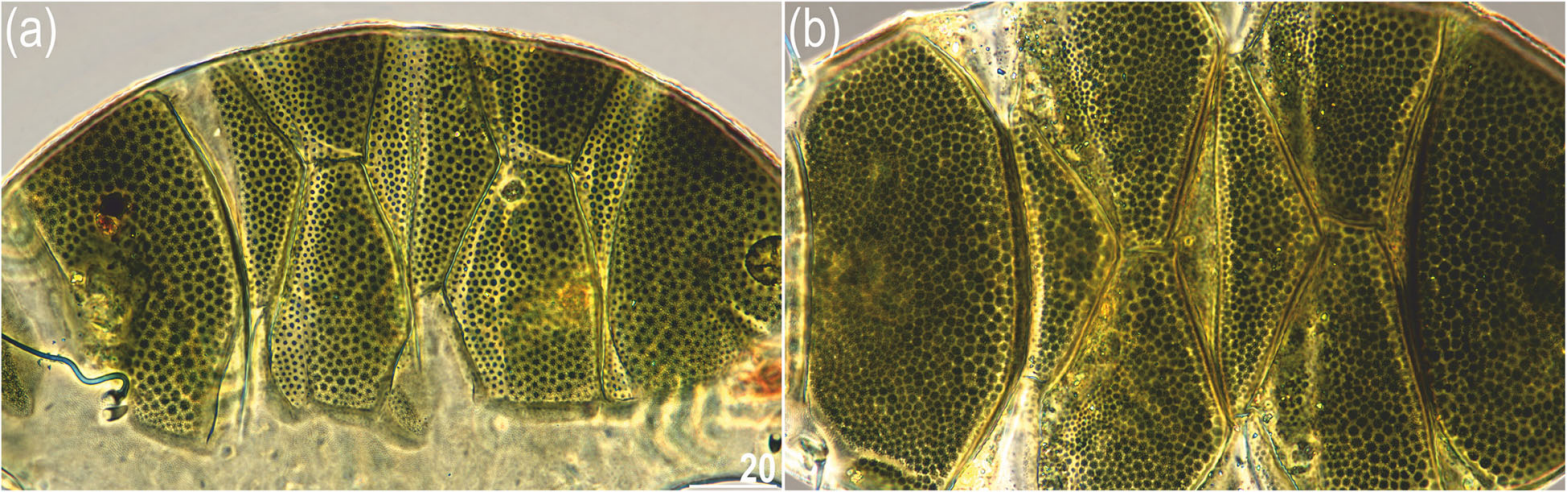
Dorsal plate sculpturing of sexually mature females of **(a)** *Viridiscus* aff. *viridianus* from North America and **(b)** *Viridiscus perviridis* from Madeira. PCM photomicrographs; scale bars in μm, both images in the same scale

**Fig. 2:**
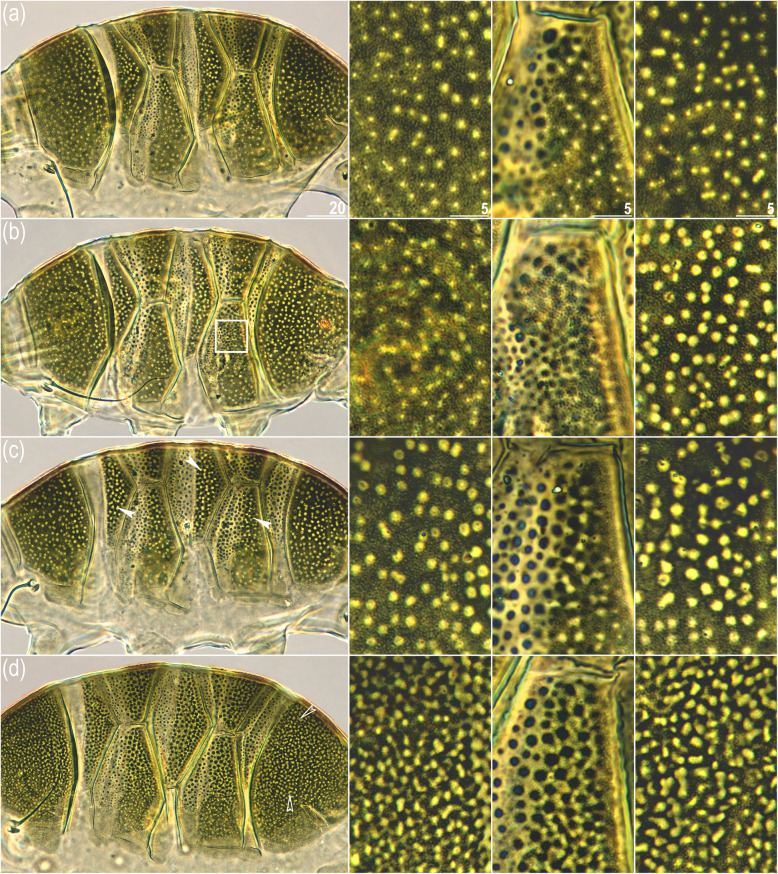
The variability (morphotypes) of dorsal plate sculpturing in *V. viridissimus*; in every row (a–d), the first column shows all dorsal plates, whereas the second, third and fourth columns show close up views of the scapular, second paired, and the caudal plates, respectively: **(a)** the most common morphotype described in the original description of the species with epicuticular granules present only in the anterior portions of the paired segmental plates, the anterior portion of median plate 2 and on the median plate 3 (the *viridissimus* morphotype; Indochina), **(b)** epicuticular granules extend toward the posterior portion of paired segmental plate 2 (indicated by the white frame) (morphotype intermediate between the *viridissimus* and *miraviridis* morphotypes; North America), **(c)** compared to morphotype “a”, epicuticular granules present on both median and paired segmental plates (indicated by filled arrowheads; intermediate morphotype; North America), **(d)** epicuticular granules dominate the dorsal armor, and 2–4 neighboring pores merge into large irregular pores, especially on the caudal plate (indicated by empty arrowheads; *miraviridis* morphotype/*V. miraviridis* **syn. nov.**; North America). PCM photomicrographs; scale bars in μm, scale in all columns the same as indicated in the first row

As in the original description of *V. viridianus*, the dorsal sculpture of *V.* aff. *viridianus* individuals from the USA (samples US.077, US.078, and US.081) was composed of densely packed epicuticular granules (Fig. 1a), whereas specimens of *V. perviridis* from Portugal (Madeira; sample PT.042) and Vietnam (samples VN.027 and VN.028) showed a similar phenotype but with a better developed endocuticular sponge layer (Fig. 1b), which is in agreement with the original description of *V. perviridis*. In both taxa, there was very little intraspecific morphological variation in the dorsal sculpturing. However, 8/21 (38%) of the analyzed specimens that otherwise fit the description of *V. viridianus* exhibited extremely long cirri *A* (50–100% of the body length), which are characteristic of *V. perviridis* (according to [17], the cirri *A* of *V. viridianus* reach a maximum length of only 20% of body length). Given these phenotypic discrepancies and the lack of available topotypic DNA sequences of *V. viridianus s.s*., we classified our specimens as *Viridiscus* aff. *viridianus*.

The American sample US.078 was also inhabited by individuals of the *V. viridissimus* (Fig. 2a) and the *V. miraviridis* (Figs. 2d) morphotypes as well as by tardigrades with two intermediate morphotypes (Fig. 2b–c). In other words, we found four morphotypes of dorsal sculpturing, constituting a *viridissimus*–*miraviridis* spectrum, where the first morphotype was attributable to *V. viridissimus*, the fourth morphotype was identifiable as *V. miraviridis*, and the two intermediate morphotypes were not classifiable as any known species. In brief, along this spectrum, the area covered with epicuticular granules increases, and adjacent round pores fuse into irregularly shaped pores (see Fig. 2 for PCM photomicrographs and a detailed description of the four morphotypes). Finally, the American (US.080 and US.081) and Vietnamese (VN.027 and VN.028) samples contained the *V. viridissimus* morphotype (Fig. 2a).

### Molecular phylogeny and species delineation (Fig. 3)

**Fig. 3:**
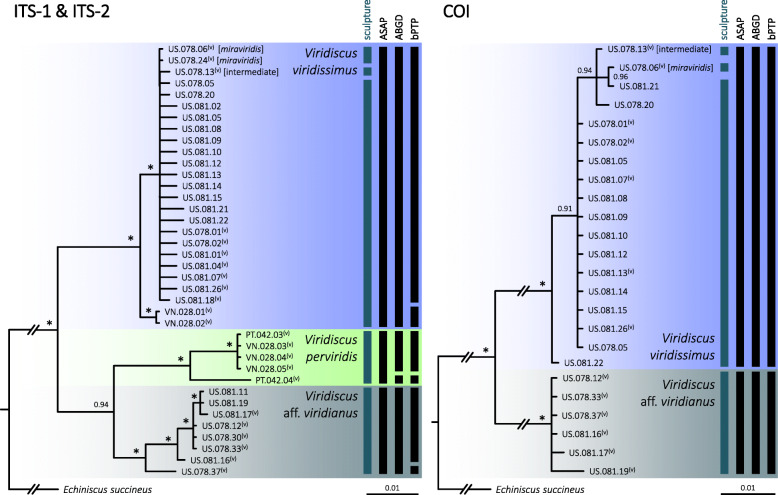
Phylogenetic relationships of the genus *Viridiscus*: **left panel:** Bayesian tree based on the concatenated ITS-1 + ITS-2 dataset (1064 bp); vertical bars denote different delineation methods used in the formulation of the primary species hypotheses: sculpture (dorsal plate sculpture observed in PCM), ASAP, ABGD, and bPTP; **right panel:** Bayesian tree based on COI (611 bp). Asterisks indicate the maximal (1.00) posterior probability value; (v) – a hologenophore was secured for post hoc PCM analysis [the *miraviridis* morphotype and an intermediate morphotype between *viridissimus* and *miraviridis* are indicated in square brackets]. *Echiniscus succineus* was used as an outgroup

In the Bayesian phylogenetic tree based on the concatenated ITS-1 + ITS-2 dataset (Fig. 3, the left panel), the 38 sequenced *Viridiscus* individuals clustered into three maximally supported clades that corresponded to the single-marker delineation ASAP models: a clade that clustered all sequences representing the *V. viridissimus*–*miraviridis* spectrum (ITS-1: five haplotypes; intraclade p-distances = 0.2–1.0%; ITS-2: two haplotypes; intraclade p-distance = 0.7%), the *V. perviridis* clade (IT1: two haplotypes; intraclade p-distance = 2.0%; ITS-2: two haplotypes; p-distance = 2.7%), and the *V.* aff. *viridianus* clade (ITS-1: five haplotypes; intraclade p-distances = 0.2–1.5%; ITS-2: three haplotypes; intraclade p-distances = 0.2–1.7%). The interclade p-distances were as follows: ITS-1: 2.5–4.0%; ITS-2: 3.7–7.8%. Another phenetic method, ABGD, favored the presence of four hypothetical species by dividing *V. perviridis* into two putative species (Fig. 3). Finally, bPTP split the haplotypes into as many as six hypothetical species, where each of the three major clades were split into two species (Fig. 3). Importantly, the specimens representing the *V. viridissimus*–*miraviridis* spectrum were divided not by morphotype but by geography (US vs. Vietnamese populations).

The Bayesian tree based on COI sequences (Fig. 3, the right panel) showed two maximally supported clades, also corresponding to the delineation analyses: the *viridissimus*–*miraviridis* clade (five haplotypes; intraclade p-distances = 0.2–0.8%) and the *V.* aff. *viridianus* clade (three haplotypes; intraclade p-distances = 0.2–1.0%). The interclade p-distances distances ranged from 14.6–15.4%.

### Integration of phenotype and genotype data (Figs. 1, 2 and 3)

We attribute the oversplitting of lineages into putative candidate species by ABGD and bPTP to the weaker performance of the two methods in comparison to ASAP [24]. Given that all sequenced individuals representing the *viridissimus*–*miraviridis* spectrum formed a single well supported but internally poorly differentiated clade (Figs. 2–3), we conclude that *V. miraviridis* is a junior synonym of *V. viridissimus*, representing a rare morphotype of the senior species.

## Discussion

The complex of species previously known as the *Echiniscus viridis* group but currently classified in the recently erected genus *Viridiscus* has always drawn the attention of tardigrade taxonomists due to the persistence of the extraordinary green body pigmentation after mounting [15–18, 20, 38]. Despite the crucial revisions by Pilato et al. [17, 18], not all of the described *Viridiscus* spp. are properly delineated. In fact, none of the species in the genus has been described or redescribed under the integrative taxonomy framework. For example, one of the key characteristics separating *V. perviridis* and *V. viridianus* is the length of cirri *A*, which greatly exceeds 50% of the body length in the former. However, we encountered single individuals of *V.* aff. *viridianus* exhibiting particularly long cirri *A* (50–100% of the body length) in the samples from Tennessee. Individuals with such long cirri may have prompted Maucci [39] to identify North American *Viridiscus* specimens as *V. perviridis*. Likewise, Nelson et al. [20] identified Tennessee specimens with long cirri as *V. perviridis*. However, given that the Tennessee specimens analyzed in this study with *perviridis*-like long cirri exhibited *viridianus*-like sculpturing, we identified them as *V.* aff. *viridianus*, together with similar specimens that have short cirri. Nevertheless, removing the uncertainty from the taxonomic identification of Tennessee *V.* aff. *viridianus* will require topotype DNA sequences of *V. perviridis* and *V. viridianus*. This illustrates the power and value of genetic data associated with type (or neotype/topotype) series and shows how problematic the lack of such data can be.

However, our study provides an even more explicit example demonstrating the importance of integrating classical methods (morphology and morphometry) and molecular tools (phylogeny and genetic delineation) for precise taxonomic inference. Although our observation of the morphological *viridissimus*–*miraviridis* spectrum itself was an indication that the validity of *V. miraviridis* was questionable, it did not allow us to determine whether the spectrum represents a single species or two closely related and interbreeding species (males of both morphotypes were found, which could favor the latter hypothesis). Interestingly, all specimens from the Maucci collection originating from Tennessee [39] present a “classical” *V. viridissimus* morphotype (such as shown in Fig. 2a). Thus, only the use of variable genetic markers, such as ITS and COI, could ultimately verify the phylogenetic position and, thus, the taxonomic identity of the observed morphotypes. Studies addressing milnesiids, possibly one of the most speciose morphologically static limnoterrestrial tardigrade lineages [40], have already emphasized that basing further descriptions of new limnoterrestrial tardigrade species solely on a morphological analysis of a small number of specimens may be detrimental to tardigrade classification [41]. Although echiniscids are the richest in taxonomically informative traits among limnoterrestrial tardigrades [11], distinguishing between intra- and interspecific variability using phenotypes alone may be unreliable and misleading [42]. The necessity of an integrative approach has also been stressed in other studies conducted on Echiniscidae (e.g., [43, 44]). Thus, the more we know about limnoterrestrial tardigrade diversity and evolution, the clearer it becomes that abandoning phenotype-based taxonomy and adopting an integrative approach is the only way to make real progress in describing and understanding tardigrade diversity, biogeography and evolution. Otherwise, we will likely face an unprecedented rate of taxonomic inflation (i.e., increases in the number of synonyms [45]), considering how much unknown tardigrade diversity likely exists and how few taxonomically useful phenotypic characters limnoterrestrial tardigrades exhibit (e.g., see recent papers addressing *Pseudechiniscus* diversity: [46–48]).

There is concern that DNA tools are not available to everyone and that they may limit the development of young taxonomists and ‘citizen scientists’, especially in developing countries. While it is true that genetic analysis entails additional costs, the price per sequenced barcode has been rapidly decreasing over the last two decades. More importantly, there are a number of laboratories around the world that are willing to provide genetic expertise through collaboration. The association of even a single variable marker, such as COI or ITS-2, with the morphological characterization of a new taxon greatly reduces the chance of taxonomic inflation without being costly in terms of effort or money. Moreover, integrative redescriptions, especially for the type species of genera and species complexes, seem more important than the description of ‘regular’ new species because poorly described type taxa often constitute a serious obstacle to elucidating the biodiversity of a given lineage (e.g., see [48] for heterotardigrades and [49] for eutardigrades). Abandoning classical taxonomy means that when it is not possible to obtain DNA data (e.g., when only old material or specimens preserved on slides are available), the description of some taxa will be postponed indefinitely until new material becomes available. However, in such cases, we need to consider whether it is more important to publish a description of a new species based solely on morphology and risk the further cluttering of limnoterrestrial tardigrade taxonomy or to wait and perform genetic analysis to advance scientific progress in the field.

Through molecular and comparative phylogenetic analyses and the integration of phenotypic and genetic data, taxonomy and systematics should gradually become more objective and more testable [50]. Fortunately, even though tardigrade species are still being described based solely on morphology, the integrative approach has become the “gold standard” since the first such study was published a decade ago [51], and the proportion of integrative works is constantly increasing [52]. Thus, hopefully by the end of this decade, journal editors and reviewers will become reluctant to accept descriptions of new limnoterrestrial taxa and, eventually, faunistic records without genetic evidence.

## Conclusions

Neither morphology nor molecular methods should be used alone to delineate tardigrade species, as this leads to for the accumulation of taxonomic issues over many decades of work. We want to raise awareness that further describing species based solely on morphology will inevitably result in serious taxonomic inflation and unreliable biogeographic data.

## Supplementary Information

**Additional file 1: SM.1** List of primers and PCR programs.

**Additional file 2: SM.2** ITS-1 nucleotide alignment.

**Additional file 3: SM.3** ITS-2 nucleotide alignment.

**Additional file 4: SM.4** COI nucleotide alignment.

## Data Availability

All data generated or analyzed during this study are included in this published article [and its supplementary information files].
